# Artificial Intelligence in Pharmacoepidemiology: A Systematic Review. Part 2–Comparison of the Performance of Artificial Intelligence and Traditional Pharmacoepidemiological Techniques

**DOI:** 10.3389/fphar.2020.568659

**Published:** 2021-01-14

**Authors:** Maurizio Sessa, David Liang, Abdul Rauf Khan, Murat Kulahci, Morten Andersen

**Affiliations:** ^1^ Department of Drug Design and Pharmacology, University of Copenhagen, Copenhagen, Denmark; ^2^ Department of Applied Mathematics and Computer Science, Technical University of Denmark, Lyngby, Denmark; ^3^ Department of Business Administration, Technology and Social Sciences, Luleå University of Technology, Luleå, Sweden

**Keywords:** systematic review, pharmacoepidemiology, artificial intelligence, machine learning, deep learning

## Abstract

**Aim:** To summarize the evidence on the performance of artificial intelligence vs. traditional pharmacoepidemiological techniques.

**Methods:** Ovid MEDLINE (01/1950 to 05/2019) was searched to identify observational studies, meta-analyses, and clinical trials using artificial intelligence techniques having a drug as the exposure or the outcome of the study. Only studies with an available full text in the English language were evaluated.

**Results:** In all, 72 original articles and five reviews were identified *via* Ovid MEDLINE of which 19 (26.4%) compared the performance of artificial intelligence techniques with traditional pharmacoepidemiological methods. In total, 44 comparisons have been performed in articles that aimed at 1) predicting the needed dosage given the patient’s characteristics (31.8%), 2) predicting the clinical response following a pharmacological treatment (29.5%), 3) predicting the occurrence/severity of adverse drug reactions (20.5%), 4) predicting the propensity score (9.1%), 5) identifying subpopulation more at risk of drug inefficacy (4.5%), 6) predicting drug consumption (2.3%), and 7) predicting drug-induced lengths of stay in hospital (2.3%). In 22 out of 44 (50.0%) comparisons, artificial intelligence performed better than traditional pharmacoepidemiological techniques. Random forest (seven out of 11 comparisons; 63.6%) and artificial neural network (six out of 10 comparisons; 60.0%) were the techniques that in most of the comparisons outperformed traditional pharmacoepidemiological methods.

**Conclusion:** Only a small fraction of articles compared the performance of artificial intelligence techniques with traditional pharmacoepidemiological methods and not all artificial intelligence techniques have been compared in a Pharmacoepidemiological setting. However, in 50% of comparisons, artificial intelligence performed better than pharmacoepidemiological techniques.

## Introduction

In the first part of this systematic review (Sessa et al., 2020), we showed that in the past decade there was increased use of machine learning techniques in Pharmacoepidemiology, which is defined by the International Society of Pharmacoepidemiology, as “*the discipline studying the utilization and effects of drugs in large numbers of people*.” In this discipline, machine learning techniques have been applied mainly on secondary data and mostly to predict the clinical response following a pharmacological treatment, the occurrence/severity of adverse drug reactions, or the needed dosage of drugs with a narrow therapeutic index. For such purposes, artificial neural networks, random forest, and support vector machine were the three most used techniques (Sessa et al., 2020). Based on such observations a natural question arise or rather “*What is the performance of machine learning when compared to traditional methods used in Pharmacoepidemiology?*” To date, a systematic evaluation of the performance of machine learning techniques in comparison with traditional pharmacoepidemiological techniques (Anes et al., 2012) is missing. Therefore, the objective of the second part of this systematic review is to provide a detailed overview of articles performing such a comparison.

## Methods

The protocol of the systematic review has been registered in the PROSPERO International Prospective Register of Systematic Reviews database (identifier CRD42019136552). The methods are described in detail in the first part of this systematic review (Sessa et al., 2020).

### Search Methods for the Identification of Studies

Ovid MEDLINE (01/1950 to 05/2019) was searched along with the references listed in the reviews identified with our research query, which is available elsewhere (Sessa et al., 2020).

### Eligibility Criteria for Considering Studies in This Review

We included observational studies, meta-analyses, and clinical trials using artificial intelligence techniques having a drug as the exposure or the outcome of the study. Only studies with an available full text in the English language were evaluated.

### Data Extraction and Management

A data extraction form was developed for this systematic review, which is available elsewhere (Sessa et al., 2020). For consistency with the first part of this systematic review, we categorized the purpose of using machine learning techniques as follows: 1) To predict clinical response following a pharmacological treatment; 2) To predict the needed dosage given the patient’s characteristics; 3) To predict the occurrence/severity of adverse drug reactions; 4) To predict diagnosis leading to a drug prescription; 5) To predict drug consumption, 6) To predict the propensity score; 7) To predict drug-induced lengths of stay in hospital; 8) To predict adherence to pharmacological treatments; 9) To optimize treatment regimen; 10) To identify subpopulation more at risk of drug inefficacy, and 11) To predict drug-drug interactions.

## Results

In all, 72 original articles and five reviews were identified *via* Ovid MEDLINE of which 19 (26.4%) compared the performance of artificial intelligence techniques with traditional pharmacoepidemiological methods. In total, 44 comparisons have been performed in articles aiming at 1) predicting the needed dosage given the patient’s characteristics (31.8%), 2) predicting the clinical response following a pharmacological treatment (29.5%), 3) predicting the occurrence/severity of adverse drug reactions (20.5%), 4) predicting the propensity score (9.1%), 5) Identifying subpopulation more at risk of drug inefficacy (4.5%), 6) predicting drug consumption (2.3%), and 7) predicting drug-induced lengths of stay in hospital (2.3%).

Compared artificial intelligence techniques included random forest (11/44), artificial neural network (10/44), decision/regression tree (7/44), support vector machine (7/44), LASSO/elasticNet (4/44), auto contractive map (1/44), Bayesian additive regression (1/44), hierarchical cluster analysis (1/44), k-nearest neighbors (1/44), and naive Bayes classifier (1/44).

In 22 out of 44 (50.0%) comparisons, artificial intelligence techniques performed better than traditional pharmacoepidemiological methods and in 4 (9.5%) cases they perform equally (Table 1). Random forest (seven out of 11 comparisons; 63.6%) and artificial neural network (six out of 10 comparisons; 60.0%) were the techniques that in most of the comparisons outperformed traditional pharmacoepidemiological methods (Table 1).

**TABLE 1:** Comparison of the performance of artificial intelligence techniques with standard pharmacoepidemiological techniques. The articles marked in red are considered potential borderline in our broad definition of pharmacoepidemiology.

Technique	Outcome 1	Outcome 2	Outcome 3	Outcome 4	Outcome 5	Outcome 6	Outcome 7	Outcome 8	Outcome 9	Outcome 10	Outcome 11
Artificial neural network	↑ ↑	↑ ↓ ↓ ↓	↓↑			↑	↑				
Auto contractive map	↑										
Bayesian additive regression trees		=									
Bayesian machine learning											
Bayesian network learning											
Classification, regression and decision tree	↓ ↓	↓ ↓ ↓	↑ ↑								
Convolutional neural network											
Decision table											
Discriminant analysis											
Fuzzy-c-means											
Hierarchical clustering			↑								
Kernel partial least squares											
K-means clustering											
K-nearest neighbors	↓										
Naïve Bayes classifier	=										
Principal component analysis											
Q-learning											
Random forest	↓ ↓ ↑	↓ ↑ =	↑ ↑		↑	↑				↑	
Ridge, ElasticNET, and LASSO	↑	↓				↑ ↑					
Support vector machine	↑ ↑	= ↓	↓ ↓							↓	

Outcome 1—To predict clinical response following a pharmacological treatment; Outcome 2—To predict the needed dosage given the patient’s characteristics; Outcome 3—To predict the occurrence/severity of adverse drug reactions; Outcome 4—To predict diagnosis leading to a drug prescription; Outcome 5—To predict drug consumption; Outcome 6—To predict the propensity score; Outcome 7—To predict drug-induced lengths of stay in hospital; Outcome 8—To predict adherence to pharmacological treatments; Outcome 9—To optimize treatment regimen; Outcome 10—To identify subpopulation more at risk of drug inefficacy; Outcome 11—To predict drug-drug interactions; ↑: artificial intelligence performed better than standard pharmacoepidemiological methods; ↓: artificial intelligence performed worse than standard pharmacoepidemiological methods; =: artificial intelligence performed equal than standard pharmacoepidemiological methods.

Below, we provided a detailed overview of the articles performing such comparisons for each of the outcomes listed above.

### Predicting the Clinical Response Following a Pharmacological Treatment

Barbieri et al. (2015) used artificial neural network and linear regression models to predict hemoglobin levels in patients with end-stage renal disease that received pharmacological treatment for anemia (e.g., darbepoetin alpha iron sucrose or iron gluconate). This observational study involved 4,135 patients undergoing hemodialysis in three different countries (Portugal, Spain, and Italy) from January 1, 2006 to December 31, 2010. Considering the heterogeneity of the study population, the authors claimed the necessity of a reliable model for the prediction of the response to antianemia therapy. Therefore, they decided to test both machine learning and the linear regression models. The artificial neural network has been modeled to mimic the human physiology by taking into account red blood cell lifespan, and schedule of drug administration (i.e., posology). The data source was electronic healthcare records of Hospital databases. The dataset used for the analysis was composed of 101,918 observations of 4,135 patients. The authors divided the dataset into two subsets. One subset composed of 66% of the data (training subset), that was used to train the models. The remaining 34% of the data was subsequently divided into two subsets containing 17% of the data each. The first subset containing 17% of the data was used for cross-validation and tuning of the models. The other was used as completely unseen data to test the performance of the models. The study outcome was hemoglobin level over time (g/dl, continuous variable). The variables used for the prediction of hemoglobin levels over time were 20 continuous variables (e.g., known predictors of hemoglobin levels). The variables codifying for the outcome and the predictors were included in the feed-forward artificial neural network and in the linear regression models. To compare the performance of both models, predicted values of hemoglobin with the observed valued were compared along with prediction errors for both models. The Mean Error (ME) was used as a measure of bias, and the Mean Absolute Error (MAE) and Root Mean Square Error (RMSE) were used as measures of accuracy the model. Prediction results showed better accuracy and reduced biased estimates of hemoglobin levels with the artificial neural network model (MAE: 0.574; RMSE: 0.764; ME: 0.010) when compared to the linear regression model (MAE: 0.610; RMSE: 0.813; ME: 0.026). According to the authors, the use of machine learning did not improve the prediction considerably. They concluded that the main reason could be that “ *a certain accuracy threshold that could not be surpassed by means of using the same data structure with different methods*.” Additionally, they supposed that the limited improvement obtained by using machine learning models “*may also be linked to the fact that the systematic error committed by measuring machines did not give a wide margin of improvement*” (Barbieri et al., 2015).

Buchner et al. (2012) compared artificial neural network and logistic regression models for the prediction of poor prognosis in a cohort of 175 patients with advanced renal cell carcinoma who started a systemic therapy for the disease (interferon alfa and interleukin-2 s. c. or oral tyrosine kinase inhibitors) between January 1, 2004 and May 31, 2009. The authors claimed that the prediction of the prognosis of patients with metastatic renal cell carcinoma is still an unresolved issue and there is a need for assessment tools (e.g., machine learning models) with improved prediction performance. According to the author, artificial neural network was a suitable technique to recognize complex data patterns in their dataset and, therefore, improve prediction accuracy of the prognosis. The data source was a dataset containing clinical measurements of patients collected in a hospital setting. The authors divided the dataset into two subsets. One subset composed of 70% of the data (training subset) that was used to train the models, and a subset containing the remaining 30% of the data that was used for assessing the prediction performance. The study outcome was overall survival after 36 months (categorical variable). The variables used to predict the outcomes were four continuous variables (e.g., age and body mass index) and 12 categorical variables (e.g., sex and type of therapy). The variables codifying the outcome and the predictors were included in the Broyden-Fletcher-Goldfarb-Shanno training algorithm to train the 3-layer multilayer perceptron model (e.g., a type of artificial neural network). For all input variables included in the artificial neural network, authors assessed a sensitivity index which indicated the classification error (e.g., misclassification of survival) if the specific variable was omitted. The authors found that the artificial neural network model outperformed the logistic regression model in prediction accuracy (95%; 166 of 175 patients) vs. 78% (137 of 175 patients)) providing a more accurate predictions of the prognosis. Furthermore, the authors compared the mean Area Under the receiver operating characteristic Curve (AUC) of the artificial neural network model (AUC: 0.952; 95% CI: 0.878–0.987) with those of the logistic regression (AUC: 0.794; 95% CI: 0.688–0.877). AUC represents degree or measure of separability or rather how much the model is capable of classifying correctly the prognosis of the patients. When the AUC was compared between the two models (*p*-value: 0.002) the artificial neural network was associated with a staitstically significant better classification performance than logistic regression (Buchner et al., 2012).

Podda et al. (2017) used a logistic regression model and several machine learning models, including the auto contractive map, random forest, naïve Bayes classifier, sequential minimal optimization (e.g., support vector machine), K-nearest neighbors, and meta bagging (e.g., decision tree) to predict the platelet reactivity (e.g., as a surrogate biomarker for the effectiveness of the treatment) in clopidogrel-treated patients. The data source was the subset of the dataset used in the GEPRESS study. The dataset contains information of 603 patients with non-ST acute coronary syndromes receiving aspirin and clopidogrel. The original dataset contained fifty-nine variables recording demographic, clinical and genetic features of patients of which 23 were evaluated by Podda et al. (2017) as potential predictors of study outcomes (e.g., platelet reactivity index and high on-treatment platelet reactivity). This included six continuous variables (e.g., age) and 17 categorical variables (e.g., sex and diabetes mellitus). The dataset was divided into a training subset (302/603) used to train the models and a testing set (301/603) used to evaluate the performance of the models. The authors found that auto contractive map (accuracy: 63%; 95% CI: 59–66%) and sequential minimal optimization models (60%; 95% CI: 53–63%) performed better than the logistic regression model (59%; 95% CI: 55–62%) in terms of accuracy of the prediction. Instead, the random forest (55%; 95% CI: 51–60%), naïve Bayes classifier (56%; 95% CI: 52–60%), K-nearest neighbors (50%; 95% CI: 46–54%) and the meta bagging models (52%; 95% CI: 48–56%) performed worse than the logistic regression model. According to the authors, even if the auto contractive map and the sequential miniminal optimizations models performed better than the logistic regression models in terms of prediction accuracy for the study outcomes, the overall prediction accuracy was not satisfactory (Podda et al., 2017).

Waljee et al. (2017) used random forest model and logistic regression models to predict clinical remission of patients with inflammatory bowel disease treated with thiopurines. The data source was electronic health records of 1,080 patients retrieved from the University of Michigan Health System Data Warehouse between November 13, 1998, and November 19, 2012. The data source was split in two subsets containing 70% and 30% of the data, respectively. These subsets have been used as a testing subset (70% of the data) and a validation subset (30% of the data). To predict clinical remission, ten variables were included in the models of which seven were categorical (e.g., sex and race) and three were continuous variables (e.g., age and disease duration). The random forest model was built by making multiple decision trees which each considered a random subset of the variables. A total of 1,000 decision trees were built and combined to build the final model. The AUC was used to evaluate the classification performance of the model. The authors found that the random forest model classified correctly patients with an AUC of 79% (95% CI: 0.78–0.81) which was significantly superior (*p*-value: < 0.05) to the logistic regression model (49%; 95% CI: 0.44–0.54). The authors discussed the potential limitations of their study. In particular, they stated that “*not all measures of objective remission were used*,” which may have contributed to a “*heterogeneity in the objective remission definition*” and therefore “*threaten the generalizability of the results*.” The authors have incorporated the random forest model into the daily clinical use at the University of Michigan with encouraging results (Waljee et al., 2017).

Sangeda et al. (2014) used multiple machine learning techniques including random forest, decision tree, Akaike information criterion stepwise logistic regression and a boost stepwise logistic regression models to predict virological failure in patients treated with antiretroviral drugs for the Human Immunodeficiency Virus (HIV). The data source was clinical data collected for 162 HIV-infected adults attending an HIV Care and Treatment Center in 2010. In the study, 17 variables were investigated as potential predictors of virological failure of which 13 were categorical and four were continuous variables. In the article, it was not described how the machine learning models were set up. The authors used AUC, sensitivity, and specificity to assess the models performance. The authors found that the random forest model had an AUC of 0.59 (SD: 0.15) which was inferior to Akaike information criterion stepwise logistic regression (AUC: 0.62, SD: 0.14) and boost stepwise logistic regression (AUC: 0.64, SD: 0.15) models. Analogously, the random forest model performed worse in term of prediction accuracy (67.80; SD: 9.11), sensitivity (0.28; SD: 0.19), and specificity (0.88; SD: 0.10) than Akaike information criterion stepwise logistic regression models [AIC stepwise: accuracy: 66.79 (SD: 10.10); sensitivity: 0.37 (SD: 0.20); specificity: 0.82 (SD: 0.12)] and boost stepwise logistic regression [accuracy: 67.77 (SD: 9.82); sensitivity: 0.39 (SD: 0.20); specificity: 0.83 (SD: 0.11)] models. Similarly, the decision tree model had an AUC of 0.55 (SD: 0.12) that was inferior to both logistic regression models (Akaike information criterion stepwise model AUC 0.62, SD: 0.14 and boost stepwise model AUC: 0.64, SD: 0.15). The decision tree model had also lower accuracy (65.72; SD: 8.46), sensitivity (0.21; SD: 0.21), and specificity (0.89; SD: 0.13) than both Akaike information criterion stepwise logistic regression [accuracy: 66.79 (SD: 10.10); sensitivity: 0.37 (SD: 0.20); specificity: 0.82 (SD: 0.12)] and boost stepwise logistic regression [accuracy: 67.77 (SD: 9.82); sensitivity: 0.39 (SD: 0.20); specificity: 0.83 (SD: 0.11)] models (Sangeda et al., 2014).

Saigo et al. (2011) investigated the association between sequences of antiviral pharmacological treatment/virus genotype changes and the occurrence of treatment failure. The data source was the EuResist integrated database which contains the treatment history (e.g., 61,831 different pharmacological treatments) of 18,467 patients with HIV from four different countries (e.g., Germany, Italy, Luxembourg, and Sweden) collected in the period 1987–2007. The database includes both continuous (the viral load measurements) and categorical (e.g., administered drugs and genotypes) variables. The authors used penalized regression (e.g., LASSO), support vector machine and logistic regression models to predict the outcome. The models included 1) drugs and mutations of the current treatment, 2) the frequencies of the drugs in past treatments, 3) the number of times a mutation occurred in previous genotypes, and 4) the number of successes and failures in the past as well as the total number of treatment changes as potential predictors for the study outcome. The analyses were performed using three different datasets: 1) patients with the number of treatment changes ≥ 10 (646 patients), ≥ 5 (1830 patients), and ≥ 1 (3,759 patients). 10-fold cross-validation was used to train, tune and test the performance of the models. In each fold, 80% of the data were used for training the models, 10% were used for tuning of the model, and the other 10% were used for the performance assessment. The results suggested that LASSO (mean of the results obtained with the three different datasets—AUC: 0.83; SD: 0.02) and the support vector machine (AUC: 0.79; SD: 0.02) had a higher value of AUC than logistic regression (AUC: 0.77; SD: 0.07). The authors emphasized the LASSO exerted its best performance especially for patients with many treatment changes (≥ 10) (Saigo et al., 2011).

Wolfson et al. (2015) used naïve Bayes classifier and Cox proportional hazard models to predict an increased cardiovascular risk in real-world data. The data source was the HMO Research Network Virtual Data Warehouse containing electronic health care and administrative data. Study subjects were selected based on enrollment into the insurance plan between January 1, 1999 and December 31, 2011. The database contained 87,363 patients. The predictors under investigation included four continuous variables (age, systolic blood pressure, cholesterol markers, and body mass index) and one categorical variable (sex). The data source was divided into two subsets. The first subset containing 75% of the data was used to train the models. The other subset containing 25% of the data was used to test the models. C-index was used to compare the models’ performance. The concordance index or C-index is a generalization of the AUC that can take into account censored data. The naïve Bayes classifier model had the same performance of the Cox proportional hazard regression model (c-index 0.79 and 0.79, respectively) for the prediction of the outcome. The authors claimed that even though the two models had the same performance, the Cox proportional hazard model assumed independence and normal distributions on covariates, which assumption “is unlikely to hold in real-world data.” According to the authors, the Cox proportional hazard model was not able to identify non-linear covariate effects and interaction in a hypothesis-free setting. The naïve Bayes classifier was instead able to overcome such limitations. However, it did not improve the prediction accuracy (Wolfson et al., 2015).

### To Predict the Needed Dosage Given the Patient’s Characteristics

Tang et al. (2017) used ANN, Bayesian additive regression trees, random forest, boosted regression tree, support vector machine, and linear regression models to predict tacrolimus dose in patients undergoing renal transplantation. The data source was electronic clinical records retrieved from two hospital databases for the time period between October 2012 and September 2014. In total, 1,045 patients were enrolled in the study population or rather stable tacrolimus-treated renal recipients with a minimum age of 18 years old. A total of 26 variables were included in the models for dose prediction of which nine variables were continuous and 14 variables were categorical. The data source was divided into a training subset (80% of the data) that was used to train the models and a test subset consisting (20% of the data) that was used to assess the performance of the models. To reduce overfitting, authors resampled 100 times the patients to be included in the two subsets. The MAEs of ANN (0.77; 95% CI: 0.66–0.88) and boosted regression tree (0.74; 95% CI: ≈ 0.65–0.83) models were higher than the linear regression model (0.73; 95% CI: 0.62–0.82). Bayesian additive regression trees (0.73; 95% CI: 0.64–0.82), random forest (0.73; 95% CI: 0.64–0.82), and support vector machine (0.73; 95% CI: 0.65–0.83) models performed similar to the linear regression model. According to the authors, machine learning models had the best accuracy in the intermediate dosing range of tacrolimus. However, it did not perform well in “*extreme dose ranges*” that following the authors’ argument “*are more needed than intermediate dosing ranges as patients in extreme dose ranges are more likely to face overdose*” (Tang et al., 2017).

Liu et al. (2015) used artificial neural network, random survival forest, regression tree, LASSO, and linear regression models to predict warfarin dosage given the patients’ clinical and demographic characteristics. The data source was the database of the International Warfarin Pharmacogenetics Consortium Cohort containing 4,798 patients treated with warfarin. In total, eight variables were used in the prediction models of which three continuous and five categorical. The data source was divided into a training subset with 3,838 randomly selected patients which is 80% of the data and a testing subset with the remaining 960 patients. ANN (MAE: 9.40; 95% CI: 8.53–10.26) the random survival forest (MAE: 9.27; 95% CI: 8.42–10.12) modes had an MAE lower than the linear regression (MAE: 9.60; 95% CI: 8.75–10.45). Regression tree (MAE: 9.75; 95% CI: 8.83–10.68) and LASSO (MAE: 9.62; 95% CI: 8.73–10.47) models had an MAE higher than the linear regression (Liu et al., 2015).

Li et al. (2015) evaluated the prediction performance of artificial neural network, random forest, boosted regression tree, support vector regression, and linear regression models to predict pharmacogenetic-guided dosage of warfarin in Chinese patients. The data source consisted of 261 patients who were recruited in a hospital between May 2011 and 2014. All patients were treated with warfarin for at least 6 weeks. Variables used for the prediction of warfarin’s dosage were categorical (genotype and age) and continuous (weight and height). The data source was divided into two subsets. The first set consisted of 80% of the patients, which was used to train the models. The remaining 20% was used to assess the performance of the models. Artificial neural network (MAE: 4.71; 95% CI: 4.23–5.19), random forest (MAE: 4.49; 95% CI: 4.02–4.96), boosted regression tree (MAE: 4.76; 95% CI: 4.27–5.25), and support vector regression models (MAE: 4.71; 95% CI: 5.56–6.40) performed worse than the linear regression model (MAE: 4.39; 95% CI: 3.94–4.84) for the prediction of the outcome. This study only contained Chinese patients and according to the authors, it is well-known limitation considering that “*Chinese population has a lower incidence of warfarin resistance*” (Li et al., 2015).

Alzubiedi and Saleh et al. (2016) used artificial neural network and linear regression models to predict warfarin dose for African-Americans patients. The data source was The International Warfarin Pharmacogenetics Consortium database. In the data source, 163 patients were African-American. Twenty-two clinical and demographical variables were included as potential predictors of warfarin dose in the models. Of those, 19 were categorical variables (e.g., diabetes, sex, and smokers) and three variables were continuous (e.g., age, height, and weight). A feed-forward neural network model with three layers was used. The artificial neural network model (MAE: 10.9) performed worse than the linear regression model (MAE: 10.8) for the outcome prediction. No confidence intervals for the MAE were provided. The authors suggested that the similar performance of the models may be due to the missing “*information of more accurate predictors of warfarin dose in the dataset*” and “*the limited sample size*” which posed limitations to the study (Alzubiedi and Saleh, 2016).

### Predicting the Occurrence/Severity of Adverse Drug Reactions

Hoang et al. (2018) used artificial neural network, gradient boosting, decision tree, support vector machine, and logistic regression models to assess if the sequences of drug prescription redemptions were predictive for the occurrence of adverse drug reactions. The data source was the Pharmaceutical Benefit Scheme in Australia in the period January 1st, 2013–December 31st, 2016. The database contains information about dispensed drugs (e.g., 7,294,244 prescriptions redemption; 728 drugs) for 10% randomly selected Australian patients (e.g., 1,807,159 patients). The authors used subsequent prescriptions to define patients as having an adverse drug reaction. In particular, if a patient redeemed a medication used to treat an adverse drug reaction (drug 2) following the administration of a medication (drug 1), the patient was defined as having an adverse drug reaction. The database used for the analyses was split into a training subset (75% of the data) that was used to train the models and a testing subset was used for the prediction (25% of the data). The performance of the models was assessed by looking at their sensitivity, specificity, positive predictive value, and negative predictive value. The positive predictive value is the probability that patients with a positive screening test truly have the condition under investigation. The negative predictive value is the probability that a patient with a negative test result is truly free of the condition under investigation. The gradient boosting model (sensitivity: 77%; specificity: 81%; positive predictive value: 76%; negative predictive value: 82%) showed the best performance for the prediction of occurrence of adverse drug reactions. Gradient boosting and the decision tree (sensitivity: 67%; specificity: 67%; positive predictive value: 65%; negative predictive value: 70%) models performed better than the logistic regression model (sensitivity: 62%; specificity: 72%; positive predictive value: 67%; negative predictive value: 68%) in terms of sensitivity, positive predictive value, and negative predictive value. Artificial neural network (sensitivity: 59%; specificity: 48%; positive predictive value: 51%; negative predictive value: 55%) and support vector machine (sensitivity: 61%; specificity: 43%; positive predictive value: 52%; negative predictive value: 52%) models performed worse than the logistic regression model in terms of sensitivity, positive predictive value, and negative predictive value. According to the authors, a major limitation of the study was the use of only medication dispensing data to detect adverse drug reactions (Hoang et al., 2018).

Jeong et al. (2018) used artificial neural network, random forest, support vector machine, and logistic regression models to predict adverse drug reactions by using laboratory test results as potential predictors. The data source was electronic health records data from a hospital in the period June 1, 1994–April 15, 2015. The database had in total data on 475,417 patients, 119,165,743 drug prescription, 34,573,581 laboratory test results, and 782,190 hospitalizations. The variables included in the statistical modes were not specified in the article. However, the authors claimed that variable prioritization was performed by using the Gini impurity index. The machine learning models were optimized using the GridSearchCV function from the sci-kit-learn library in *Python* and tenfold cross-validation. The performances of the models ware compared by using sensitivity, specificity, positive predictive value, negative predictive value, F score, and AUC. Artificial neural network (sensitivity: 0.793 ± 0.062; specificity: 0.619 ± 0.061; positive predictive value: 0.645 ± 0.047; negative predictive value: 0.777 ± 0.052; F score: 0.709 ± 0.037; AUC: 0.795 ± 0.034) and random forest models (Sensitivity: 0.671 ± 0.054; specificity: 0.780 ± 0.046; positive predictive value: 0.727 ± 0.050; negative predictive value: 0.732 ± 0.043; F score: 0.696 ± 0.041; AUC: 0.816 ± 0.031) performed better than the logistic regression model (sensitivity: 0.593 ± 0.063; specificity: 0.756 ± 0.047; positive predictive value: 0.679 ± 0.048; negative predictive value: 0.682 ± 0.049; F score: 0.631 ± 0.047; AUC: 0.741 ± 0.041). Support vector machine (Sensitivity: 0.569 ± 0.056; specificity: 0.796 ± 0.046; positive predictive value: 0.709 ± 0.053; negative predictive value: 0.680 ± 0.043; F score: 0.629 ± 0.045; AUC: 0.737 ± 0.040) instead, performed worse than the logistic regression model. According to the authors, each machine learning techniques showed differences in performance indexes (Jeong et al., 2018).

Molassiotis et al. (2012) used random forest, hierarchical cluster analysis, and linear regression models to cluster signs and symptoms that could predict the occurrence of nausea in patients receiving chemotherapy. The data source was primary data collected from a prospective cohort study of 104 patients aged ≥ 18 that received two cycles of chemotherapy in a hospital setting. Thirty-two categorical variables (e.g., dry mouth, vomiting, and itching) were included in the models for the prediction of the study outcome. The data source was split into two subsets, one used to train the models (63% of the data) and one used to assess the performance of the models (37% of the data). According to the authors, the random forest model and hierarchical cluster analysis outperformed the linear regression model in terms of prediction accuracy (results not provided). The authors highlighted potential limitations of their study by providing the disclaimer that “the study population was composed predominantly of breast cancer patients receiving anthracyclines.” Therefore, the generalizability of the findings is limited to this population of patients (Molassiotis et al., 2012).

### To Predict Drug Consumption

Devinsky et al. (2016) used random forest and logistic regression models to predict treatment changes (new, add-on or switch) in patients with epilepsy. The data source was medical, pharmacy, and hospital electronic healthcare records of 34,990 patients from the American medical claims database in the period January 1, 2006–September 31, 2011. The study evaluated 5,000 potential predictors of treatment changes, which were reduced to two continuous variables (age and number of antiepileptic drugs) and four categorical variables (sex, region, antiepileptic drugs at index date and physician prescribing the treatment) after variables prioritizations. The proportion of testing and training subsets was not stated. The random model outperformed in terms of AUC (AUC: 0.715) the logistic regression model (AUC: 0.598). According to the authors, their “model’s recommendation system could reduce treatment changes and save substantial costs while providing more stability and better outcomes to patients.” Additionally, they claimed that “future studies should evaluate other populations/databases, other therapies (e.g.,, diet, surgery, neurostimulation), as well as models with more specific outcome measures (e.g.,, seizure frequency) and perhaps even prospectively collected electronic medical records” (Devinsky et al., 2016).

### To Predict the Propensity Score

Setoguchi et al. (2008) used artificial neural network and logistic regression models to predict propensity score in two simulated clinical scenarios. The datasets, one containing 2,000 subjects and the other containing 10,000 subjects, were simulated through Monte-Carlo simulations. Both datasets contained categorical variables codyfing for binary exposures and binary outcomes. Furthermore, six categorical and four continuous covariates were simulated in both datasets. The comparison of the models’ performance was performed using the c-index. When compared to the logistic regression model (c-index 0.76, standard error 0.38; bias of the stimate 6.29%), the artificial neural network model (c-index 0.86, standard error 0.40; bias of the stimate 6.57%) provided less biased estimates of the propensity score in simulated scenarios (e.g., estimates not provided). According to the authors, the simulated data were realistic, however, “further studies are needed to assess the usefulness of data mining techniques in a broader range of realistic scenarios” (Setoguchi et al., 2008).

Karim et al. (2018) compared the performance of LASSO/elasticNET regressions and random forest models for variable prioritization before logistic regression (e.g., hybrid approaches) and logistic regression only for the computation of high-dimensional propensity score. According to the author, this approach was needed because to date, many uninformative variables are considered for the estimation of the high dimensional propensity score causing increased complexity of statistical modeling and long computational time. The data source was simulated data containing 500 variables codifying for an hypothetical exposure and outcome, and demographic and clinical characteristics of the patients. The 500 variables underwent prioritization as previously described. The prioritized variables were subsequently used for the computation of the high dimensional propensity score. The propensity score was used for statistical adjustment for the computation of the risk estimate for the simulated outcome between the two exposure levels. To evaluate the model performance, the authors used the risk difference between the estimated risk from the models and the true risk. In particular, they considered a risk difference of zero as the unbiased estimate. In presence of unmeasured confounding (bias-based analysis) and covariate effect multiplier of five, Hybrid-ElasticNet (risk difference 0.39), random forest (risk difference 0.51), and Hybrid-LASSO (risk difference 0.38) approaches provided a less biased estimate than logistic regression only (risk difference 0.55). According to the authors, “Random forest, Hybrid-ElasticNet, and Hybrid-LASSO, that further refined the confounder selection from a chosen high-dimensional propensity score selected variable pool, performed better than the regular high-dimensional propensity score approaches performed with logistic regression.” However, several limitations were identified. In particular, according to the authors, “LASSO tends to select only one variable from a group of multicollinear variables and ignores the rest of them.” They further claimed that “the exclusion of collinear variables could potentially result in residual confounding.” In this regard, the authors found beneficial the use of Elastic-net regression “more stable than a LASSO even in the presence of severe multicollinearity” (Karim et al., 2018).

### To Predict Drug-Induced Lengths of Stay in Hospital

Kim et al. (2000) used ANN and logistic regression models to predict the length of stays in the post-anesthesia care unit following general anesthesia. The data source was retrospectively collected data of 592 patients aged 16 or above undergoing general anesthesia in a hospital setting in the period March 1998–June 1998. In total, 22 variables were used for training the models of which two were continuous (age and duration of anesthesia) and 20 were categorical (e.g., sex, electrolyte imbalance, and operation site). The ANN model showed a better classification accuracy (149/183 patients correctly classified) than the logistic regression model (119/183 patients correctly classified). According to the authors, a major limitation of the study was the small sample size (Kim et al., 2000).

### To Identify Subpopulation More at Risk of Drug Inefficacy

An et al. (2018) used support vector machine, random forest, and linear regression models to predict drug-resistant epilepsy among patients treated with antiepileptics. The data source was administrative data of 292,892 patients from IQVIA databases in the period January 1st, 2006–December 31st, 2015. The data included hospital admission/hospitalization and prescription redemptions from pharmacies. In total, 1,270 variables were extracted from the database which codifies for demographics characteristics, comorbidities, insurance policy, treatments, or encounters of patients. The database was first divided into three subsets with ratios 60%/20%/20% of the data. The subset containing 60% of the data was used to train the models. The other two subsets were used for validation/calibration and performance assessment (testing). Evaluation of the models' performance was done by using the AUC. The support vector machine and the random forest models were built using 50% (635 variables) of the variables with the highest predictive value for the outcome. The support vector machine and the random forest models had an AUC of 0.745 (95% CI: 0.740, 0.751) and 0.764 (95% CI: 0.759, 0.770) respectively, whereas the multivariate linear regression model had an AUC of 0.748 (95% CI: 0.742, 0.753). This indicates that the random forest and the support vector machine performed better and worse respectively than the linear regression model. The major limitation of the study was related to the follow-up period of the patients that were on average two years. According to the authors, a major study limitation is the short follow-up period of the patients (e.g., on average two years) considering that “*it usually takes longer than two years for a patient to became drug-resistant to the antiepileptic treatment*” (An et al., 2018).

### To Predict Diagnosis Leading to a Drug Prescription; to Predict Adherence to Pharmacological Treatments; to Optimize Treatment Regimen; to Predict Drug-Drug Interactions

None of the retrieved articles compared artificial intelligence techniques with traditional pharmacoepidemiological methods for the aforementioned outcomes.

## Discussion

To the best of our knowledge, this is the first systematic review summarizing available evidence on the performance of artificial intelligence techniques vs. traditional pharmacoepidemiological techniques in a pharmacoepidemiological setting. Only 26.4% of retrieved articles used both approaches and, in several articles, it was not clearly described how the models were built and/or trained before the assessment of the prediction performance. This phenomenon is not new, considering that several concerns arose after the burst of artificial intelligence in biomedical fields, which include the risk of poor transparency/reproducibility of the results related to the incorrect reporting of artificial intelligence prediction models (Collins and Moons, 2019). We strongly believe that when artificial intelligence techniques are used, the deriving results should be replicated using traditional techniques as also suggested by Collins et al. (Collins and Moons, 2019). It is crucial that if researchers use artificial intelligence techniques, they should adhere to the established standards for reporting as currently done with traditional pharmacoepidemiological methods. It cannot be excluded that pharmacoepidemiologists by using artificial intelligence techniques may perform errors such as over predictions, overfitting, or non-optimal assessment of the prediction accuracy (Chen and Asch, 2017) that can be identified by experts during peer-review if the methods used are properly reported. It should be mentioned that a formal guideline on the topic has been released in 2015 (Collins et al., 2015).

It should be noted that for many outcomes commonly assessed in pharmacoepidemiology (e.g., adherence to pharmacological treatments and drug-drug interactions), a comparison between artificial intelligence techniques and traditional pharmacoepidemiological methods is not performed. Additionally, many promising artificial intelligence techniques (e.g., LASSO/elasticNet, auto contractive map, Bayesian additive regression, hierarchical cluster analysis, k-nearest neighbors, and naive Bayes classifier) have been compared scarcely highlighting areas for which further research is needed. In cases for which such a comparison was performed, artificial intelligence techniques exerted heterogeneity of performance with random forest and ANN being the techniques that in most cases outperformed traditional pharmacoepidemiological methods. It cannot be excluded that this result is due to publication bias. Similarly, the analytical advantage of using artificial intelligence techniques in terms of prediction accuracy cannot be overlooked either. In that sense, the artificial intelligence methods undoubtedly represent an important class of tools to improve individual care and to promote innovation in medical research (Chen and Asch, 2017).

### Strenghts and Limitations

The main strength of this systematic review is the extensive and systematic overview of currently used machine learning techniques in Pharmacoepidemiology. To the best to our knowledge, this has been performed for the first time in the scientific literature. Additionally, the extensive evaluation of each study in terms of which techniques have been compared and the outcome of these comparisons are provided. The main limitation is the unavoidable risk of publication bias which may have led to include more articles with the superior performance of machine learning techniques over traditional pharmacoepidemiological methods. Another limitation is the eclectic description of the findings in each study driven by the fact that only a few articles performed a statistical test to evaluate differences in the performance of the models under evaluation. In this regard, we relied on the point estimates of measures used to evaluate the performance of the models for claiming superiority, equity, or inferiority of artificial intelligence techniques and traditional pharmacoepidemiological methods.

## Conclusion

Only a small fraction of articles compared artificial intelligence techniques with traditional pharmacoepidemiological methods despite the recommendations from experts. Such comparisons have been not performed for many outcomes routinely assessed in pharmacoepidemiology as in the case of adherence and drug-drug interaction. In half of the comparisons, artificial intelligence performed better than traditional pharmacoepidemiological techniques with high heterogeneity in the performance among different artificial intelligence techniques. Many techniques have been scarcely investigated. Together, these results suggest that further research is needed focusing on head-to-head comparisons of traditional pharmacoepidemiological techniques with machine learning techniques in different research scenarios and with a variety of different data sources.

## Author Contributions

MS: developed the concept and designed the systematic review. MS and DL: performed the analysis and interpretation of data. DL, AK, MK, and MA: drafted the paper and revising it for important intellectual content. MS, DL, AK, MK, and MA: wrote the paper. DL, AK, MK, and MA: gave the final approval of the version to be published.

## Funding

MA professorship is supported by a grant from the Novo Nordisk Foundation to the University of Copenhagen (NNF15SA0018404). MS is supported by a grant from Helsefonden (20-B-0059).

## Conflict of Interest

DL, MA, and MS belong to the Pharmacovigilance Research Center, Department of Drug Design and Pharmacology, University of Copenhagen. DL has been an employee (e.g., a student assistant) of Alcon and Novartis in the period 2017–2019. MA reports grants from Novartis, grants from Pfizer, grants from Janssen, grants from AstraZeneca, grants from H. Lundbeck and Mertz, grants from Novo Nordisk Foundation, outside the submitted work; and personal fees from Medicademy, the Danish Pharmaceutical Industry Association, for leading and teaching pharmacoepidemiology courses.

The remaining authors declare that the research was conducted in the absence of any commercial or financial relationships that could be construed as a potential conflict of interest.
